# A Combined Approach to Heat Stress Effect on Male Fertility in *Nasonia vitripennis*: From the Physiological Consequences on Spermatogenesis to the Reproductive Adjustment of Females Mated with Stressed Males

**DOI:** 10.1371/journal.pone.0120656

**Published:** 2015-03-25

**Authors:** Marlène Chirault, Christophe Lucas, Marlène Goubault, Claude Chevrier, Christophe Bressac, Charlotte Lécureuil

**Affiliations:** Institut de Recherche sur la Biologie de l’Insecte, UMR 7261, Université François-Rabelais / CNRS, UFR Sciences et Techniques, Parc Grandmont, Tours, France; Inserm, FRANCE

## Abstract

In recent years, several studies have shown a decline in reproductive success in males in both humans and wildlife. Research on male fertility has largely focused on vertebrates, although invertebrates constitute the vast majority of terrestrial biodiversity. The reduction of their reproductive capacities due to environmental stresses can have strong negative ecological impacts, and also dramatic consequences on world food production if it affects the reproductive success of biological control agents, such as parasitic wasps used to control crop pests. Here *Nasonia vitripennis*, a parasitic wasp of various fly species, was studied to test the effects of 24h-heat stress applied during the first pupal stage on male fertility. Results showed that only primary spermatocytes were present at the first pupal stage in all cysts of the testes. Heat stress caused a delay in spermatogenesis during development and a significant decrease in sperm stock at emergence. Females mated with these heat-stressed males showed a reduce sperm count stored in their spermatheca. Females did not appear to distinguish heat-stressed from control males and did not remate more frequently to compensate for the lack of sperm transferred. As a result, females mated with heat-stressed males produced a suboptimal lifetime offspring sex ratio compared to those mated with control males. This could further impact the population dynamics of this species. *N*. *vitripennis* appears to be an interesting biological model to study the mechanisms of subfertility and its consequence on female reproductive strategies and provides new research perspectives in both invertebrates and vertebrates.

## Introduction

In recent decades many scientific studies have reported a decrease in male reproductive success linked to environment changes [1] such as a reduction in offspring number, a modification of sex ratio, or a reduction in offspring viability [2–5]. Several studies have reported that direct effects on semen quantity or quality can be in part responsible for this decrease. In men, a meta-analysis of 61 studies showed a decrease in sperm count of nearly 50% between 1940 and 1990 [6]. A declining sperm count is also found in wildlife. For example, in fish, reptiles and turtles, a series of reproductive abnormalities including lower sperm counts and an alteration of gonadal morphology and hormone concentrations have been observed in populations living in polluted areas [7–10]. Research on male fertility has largely focused on vertebrates, but invertebrates constitute the vast majority of terrestrial biodiversity [11] and reducing the reproductive capacities of species of agronomic importance, such as pollinators and biological control agents can have negative consequences on world food production. In many insect species, impaired fertility is one of the main causes of population decline, because females of many social insect species in the Hymenoptera order store sperm in a spermatheca after a single mating for several weeks, or even years in the case of bees [12,13].

Hymenoptera is the most diversified insect group which includes bees, wasps and ants. Parasitoid wasps represent an ecological and agronomic interest through their use to control invasive species or agricultural pests naturally. Parasitoid females lay their eggs in/on an insect host which is then consumed by the developing larva resulting in host death. To control pest populations efficiently, the number of laying parasitoid females present in the field is therefore crucial. Parasitic wasps are also ideal models to study functional fertility of males because of their haplo-diploid sex determination. Males are haploid and arise from unfertilized eggs, while females are diploid and arise from fertilized eggs [14]. The female ability to produce daughters, and consequently the F1 population dynamics, depends on the availability of sperm stored in the spermathaeca after mating and successful insemination. *Nasonia vitripennis* is emerging as a new insect model system because of its short generation time (2 weeks), and simple cheap, easy and productive laboratory rearing. Extensive ecological [15], developmental [16], genetic [17] and behavioral [18–20] knowledge exists for this species and its genome has recently been sequenced [21] and functional genomic tools developed [22]. It is a parasitic wasp of different flesh fly species occurring in nests of hole-breeding birds. Hosts are patchily distributed and brachypterous males are flightless and relatively short lived. Therefore, mating typically occurs immediately after emergence at the natal site. Mating occurs after a stereotypic courtship sequence during which the male elicits female receptivity [23,24]. As females typically mate only once with the first male they encounter [25], it is crucial for a courting male to attract other virgins to his vicinity. To do so, males produce a sex pheromone in their abdomen, (4R,5S)- and (4R,5R)-5-hydroxy-4-decanolides (HDL), which is released into the substrate via an anal orifice after copulation. Only virgin females respond to the pheromone [26,27]. Males able to produce greater amounts of pheromone therefore should have better chances of mating. After copulation only females disperse to new host patches.

Due to their small size and ectothermic physiology, insects are especially vulnerable to environmental changes, in particular temperature has a direct effect on behavior (e.g. flight, oviposition, courtship and mating), and fitness (e.g. gametogenesis, fecundity, developmental rate and lifespan) [28]. Damage caused by temperature changes, such as alterations in development [29], has been investigated in different insect species. Although less is known about the impacts of sub-lethal thermal stress than about lethal consequences, the temperature-development relationship has been shown to be approximately linear, increasing progressively to a maximum level before decreasing on approaching lethality [30]. Despite surviving exposure to heat stress, the reproductive potential of the survivors could be affected, for example resulting in a decrease in egg fertilization or a reduction in male fertility [31–35]. Studying the physiological responses of insects to thermal stress for each function separately is important to understand the mechanisms involved in thermal stress and could help to forecast effects of climate warming. Furthermore, it seems relevant to focus on fertility as reproductive processes are often affected by less severe conditions than those affecting survival and may therefore be evolutionarily more relevant [36].

In *Anisopteromalus calandrae*, a solitary parasitic wasp with continuous spermatogenesis, males that have been heat-stressed during the first pupal stage, have fewer spermatozoa at emergence than control males [5]. In contrast, in *N*. *vitripennis*, spermatogenesis has been suggested to be synchronized, probably due to the males’ short lives and because most matings occur immediately after male emergence [37]. Thus optimal conditions require most of the cysts within a testis to be at the same or similar developmental stages in maturing pupae. We would therefore expect heat stress to have a more dramatic effect on sperm production in *N*. *vitripennis* due to its direct impact on germ cells and to the synchronized spermatogenesis.

This study therefore aimed first to investigate the consequences of heat stress applied during the early pupal stage on *N*. *vitripennis* males (emergence, survival rates, spermatozoa stock and reproductive behavior). Secondly, it aimed to confirm synchronized spermatogenesis and determine the state of germ cells at the beginning of the pupal stage before analyzing the consequences of heat stress on spermatogenesis. The third objective was to explore some potential ecological consequences of male heat-stress induced subfertility. This research thus intended to answer the following questions: Can females recognize heat-stressed males and avoid mating with them? Are females constrained by the reduced sperm stock received from heat-stressed males? Is the sex-ratio of offspring affected? The possible subsequent impacts on the population dynamics of this species are discussed.

## Materials and Methods

### Rearing conditions

Strain AsymC of the parasitic wasp *Nasonia vitripennis* (Hymenoptera: Pteromalidae) was obtained from the Evolutionary Genetics laboratory (Groningen, The Netherlands). Insects were reared at 25°C under constant light and passive humidity in a climate room and developed on fresh *Calliphora sp* (Diptera: Calliphoridae) pupae (for details see [16]). Generation time under these conditions is approximately 13 days for males and 14 days for females. To obtain virgin parasitoids of either sex, virgin females were isolated by excising pupae from host puparium one to four days prior to emergence and keeping them in a 2 mL microcentrifuge tube (Sarstedt, Germany). After females emerged, hosts were placed in another tube to obtain virgin males.

### Stress treatments

Heat stress was applied to the first pupal stage with uncolored eyes (9 days after female oviposition, thereafter called white pupae). Male pupae were divided into 2 groups: (1) male pupae were maintained in the climate room at 25°C until emergence (= control males), or (2) male pupae were heat-treated, at 34, 36 or 38°C, for 24h in a climate-controlled incubator then returned to the climate room at 25°C until emergence (= heat-stressed males). This range of temperatures was used, because it has already been used to investigate rearing and stress in *A*. *calandrae* [5]. As *N*.*vitripennis* develops faster the duration of the treatment was reduced to 24h.

### Development

For each treatment, the development period from egg eclosion until emergence of adults, the percentage of individuals surviving to adulthood and the longevity of adult males were recorded.

### Sperm counts

The reproductive tracts of virgin males and mated females were dissected in a drop of saline solution (128.3mM NaCl; 4.7mM KCl; 2.3mM CaCl_2_). Sperm was extracted and spermatozoa counted from the seminal vesicles of virgin males at several ages and also from the spermatheca of newly emerged females isolated 24h after a single mating by carefully opening them with minuten pins. Sperm was fixed with ethanol, air-dried then stained with 4’,6-diamidino-2-phenylindole (DAPI; 2.10^−6^ g.mL^−1^) for 15 min. Sperm nuclei were counted under a fluorescence microscope (Olympus BX51, Japan) [38].

### Male courtship behavior and female mate choice

Newly emerged control or heat-stressed males were provided with one newly emerged virgin female in a 2 mL microcentrifuge tube. The length of the different stages of courtship behavior (pre-copulatory behavior, copulation and post-copulatory behaviors as previously described [18]) were recorded under a binocular microscope. The behavioral responses of virgin females to male pheromones were tested in a still-air Y-tube olfactometer made of translucent polyethylene (Kartell, Italy) [39]. The Y-tube was tilted horizontally on a piece of white cardboard and maintained at 25°C. After copulation with a newly emerged female in a 0.5 mL microcentrifuge tube (Trefflab, Switzerland), control and heat-stressed 2-day-old males were placed in chambers fixed at the end of each arm of the Y-tube for five minutes before the experiment started. Virgin females were then individually released into the olfactometer via the stem (75 mm long and inner diameter of 5 mm), and allowed to choose one of the two arms (30 mm long and inner diameter of 5 mm) which for the first experiment contained no male or one control male, and for the second experiment, one heat-stressed male or one control male. Females were considered to have made a choice when they had walked at least 2.5 cm into one of the two arms. All individuals and the olfactometer apparatus were used only once for each bioassay.

### Female remating rate

The number of newly emerged females that remated with a virgin male when presented with a second male 24 hours after the first copulation and oviposition was recorded. If no copulation occurred after three male copulation attempts, the females were considered as not willing to remate.

### Pheromone quantifications

Male pheromone titrations were carried out using organic solvent extraction and analyzed by Gas Chromatography coupled with Mass Spectrometry (7890 GC System, 7000C GC/MS triple quad, Agilent technologies, USA). Just after a single mating with a newly emerged female, 2-day-old control and heat-stressed males were individually placed in a microvial glass (Interchim, France). After five hours, males were extracted twice with 100 μL of Heptane (Sigma Aldrich, USA) and 2 min of vortex agitation. Extracts were then transferred into a new tube containing 4 ng of eicosane (C20) (Sigma-Aldrich, USA) as an internal standard and stored at −20°C. For analysis, samples were evaporated under a gentle nitrogen flow and diluted into 2 μL of solvent just prior to manual injection. Chemical analysis was carried out using a GC-MS fitted with a HP-5MS fused silica capillary column (30m x 0.250 mm x 0.25 μm film thickness; HP-5 MS UI, Agilent, USA) with an electron impact of 70eV (scanning mode 40–550 amu) in splitless mode with a splitless time of two minutes at 250°C. The oven program began at 100°C then ramped at 3°C/min to 120°C, then 30°C/min to 200°C, and 5°C/min to 320°C (held 5 min). The carrier gas was Helium at a flow rate of 1 mL/min. The pheromone components (4*R*,5*R*)- and (4*R*,5*S*)-5-hydroxy-4-decanolides (HDL) were identified using fragmentation analysis [40,41] with Masshunter software (qualitative analysis B.06.00, Agilent technologies) and compared to published data [42,43]. Quantification were done by calculate relating peak areas to the internal standard. Quantities were calculated by relating peak areas to the internal standard

### Sex ratio

After a single mating with either a newly emerged control or heat-stressed male, zero to one day old females were individually placed in a box (Solo no.P100, USA) with 2 fresh hosts, and a cotton ball soaked in 40% (w/v) sugar solution which was changed every 24h. Hosts were kept at 25°C until the sex of the offspring was determined visually at the pupal stage after dissection of the host puparium.

### Histology

Histological analyses of the abdomen of first pupal stage males (i.e. at the time the heat stress was applied), second pupal stage of control and heat-stressed males (i.e. just after heat stress) and final pupal stage of control and heat-stressed males (i.e. 72 hours after heat stress), were processed using the protocol described by [44]. In brief, males at several pupal stages were dissected rapidly under a fixative (2.5% glutaraldehyde and 2.0% para-formaldehyde in phosphate buffer, at pH 7.3) and stored in the same solution for about three hours. After dehydration, they were embedded in Durcupan ACM (Electron Microscopy Sciences, Hatfield, PA, USA) via propylene oxide. Blocks were serially sectioned at 2μm using glass knives mounted on a microtome (Leica RM2265, Germany). The sections were stained on a hot plate with Toluidine Blue-Basic Fuchsin and mounted on a slide with DPX (Electron Microscopy Sciences).

### RNA extraction and RT-PCR

Total RNA was extracted from 20 control male white pupae using a commercial kit (Nucleospin RNA XS, Macherey-Nagel, Duren Germany) according to the manufacturer’s protocol for tissue. Reverse transcription was performed with 500 ng of total RNA determined by a spectrophotometer (Varian, Cary 50). An oligodT primer (Promega, USA) was used with Omniscript Reverse transcriptase (Qiagen, Germany) following the manufacturer’s instructions. The cDNAs obtained were stored at −20°C. PCR reactions were performed on an ABI Prism 7000 (Applied Biosystems) in a total volume of 12.5μL with 3.125μM of forward and reverse primers (Eurofins MWG Operons, Ebersberg Germany, Table 1). The PCR products were separated on a 2% agarose gel with 0.002% Gel Red (Biotium, Hayward), and compared with a molecular weight marker (Smart Ladder SF, Eurogentec).

**Table 1:** PCR primers for *Nasonia vitripennis* gene expression analysis.

Gene	GenBank Acc.No	Forward	Reverse	mRNA Product (bps)
**Pumilio**	XM_001599192.2	AAATCAGGTGGGTTCTAGAGTTC	GCCTGAGTGCCTCGTTAAA	98
**Vasa**	XM_001603906.2	ACCAGTGCACCAGTAAATGAA	AGTGCGACTATGGTCATCAAAT	99
**Rec8**	XM_003423789.1	TCAGCAACTCCATCAGCATC	GGGAAGATTCTTTCGTCCTTGTA	130
**Spo11**	XM_001599527.1	TTATGTGCGTCTACAGATTCGG	ATTGTACAGACGCAAGTCTTCT	69
**Tektin**	XM_001599550.2	TCGATTGCGCCAAATCAAC	GTCGTCAAAGCGTGTTTCTTAT	91
**Rpl7a**	NM_001159854.1	AAGAAAGTCGAGCCCAAGAAG	GGCTGAATATCCTGGCCAAT	83

### Statistical analyses

Survival analyses with a Weibull distribution were used to compare development time and adult longevity between heat-stressed and control males. To compare sperm counts after different heat stresses, a Kruskall-Wallis test was performed, followed by 2-by-2 Mann-Whitney tests. The effects of heat stress on the reproduction parameters (sperm counts in seminal vesicles, pheromone titers, courtship time, and offspring sex ratio) were analyzed using Mann-Whitney *U*-tests because original data were not normally distributed. Statistical analyses were performed using the statistical package R (version 3.0.2).

## Results

### Consequences on male functions of heat stress applied during development

**Fig 1:**
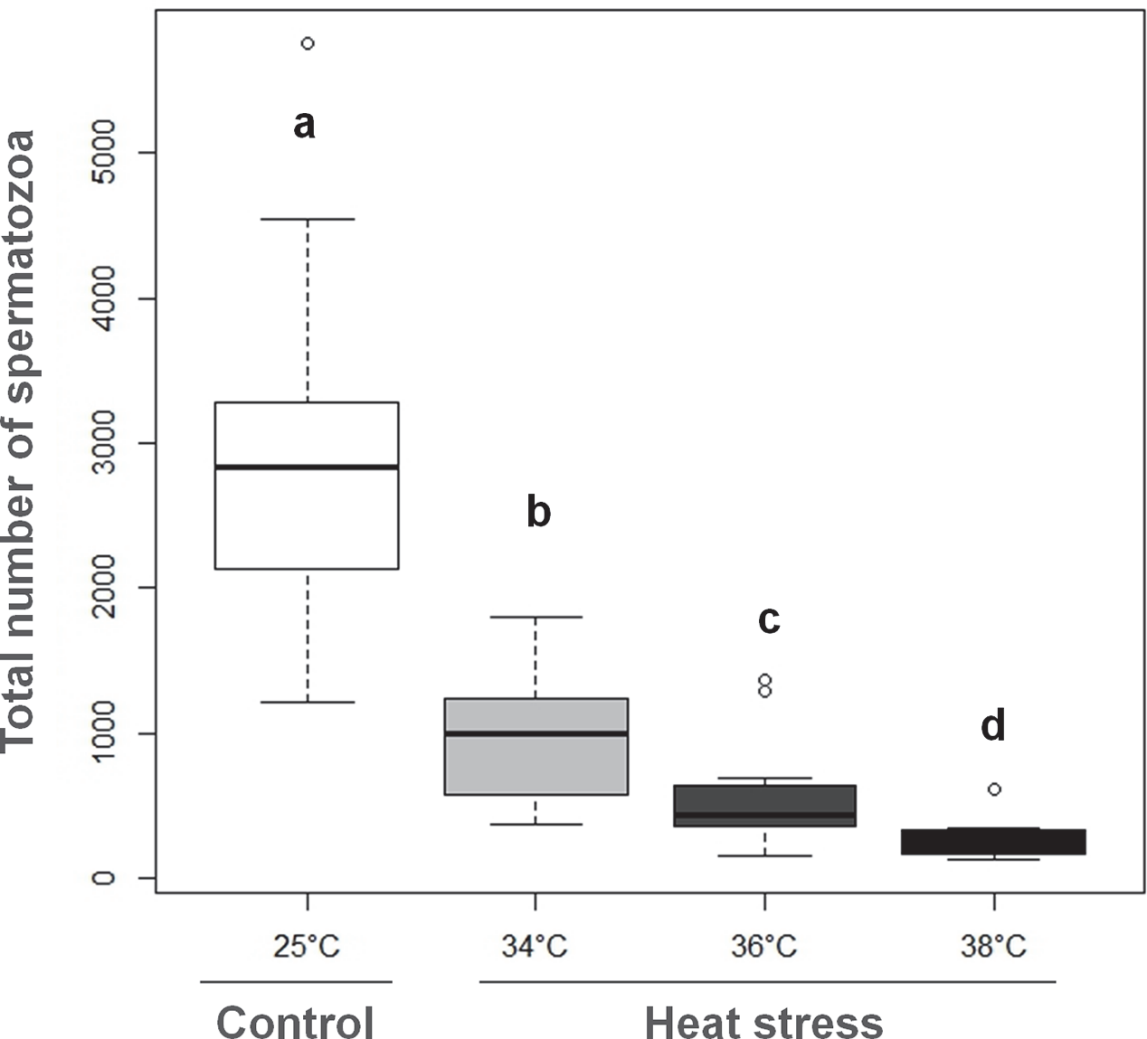
Sperm stock in seminal vesicles of emerging N. vitripennis white pupa males stressed at 34, 36 & 38°C for 24 hours (median with lower and upper quartiles). Different letters indicate a significant difference between treatments at α = 0.05 (25° C: N = 12, 34°C: N = 11, 36°C: N = 11 and 38°C: N = 10).

Exposure of the first pupal stage to high temperature did not affect the proportion of emerging adults (Table 2). Nevertheless, after heat stress at 38°C the development time increased significantly by 1.4 days and adult longevity was significantly reduced by 1.4 days. Following the exposure of white pupae to 34, 36 or 38°C during male development, sperm was counted in the seminal vesicles of males at their emergence. Control males had 2,953 spermatozoa on average, whereas for heat-stressed males a significant temperature-dependent reduction in numbers was noticed (K = 33.23, P < 0.001; Fig. 1). Compared to control males, the number of sperm in the seminal vesicles of heat-stressed males was reduced by 67% at 34°C, 81% at 36°C, and 91% at 38°C. Consequently, to evaluate the effect of heat stressing pupae on spermatogenesis and male reproductive success, it was necessary to select a temperature which caused a significant reduction in spermatozoa in seminal vesicles, while allowing successfully emergence as adults without modifying the development time or survival rate. On the basis of the above data, we chose to work with a heat stress of 36°C for 24 hours, since this treatment reduced the number of spermatozoa in the seminal vesicles at emergence by 81% compared to controls, without modifying development or survival.

**Table 2:** Effects of different heat stresses, applied on white pupae, on adult emergence and survival of *N*. *vitripennis*.

Treatment	Emergence rate (%)	Development time (days)	Longevity (days)
**25°C Control**	96.7 (N = 30)	13.2 ± 0.07 (n = 29)	6.9 ± 0.2 (n = 29)
**34°C Heat-stressed**	80.0 (N = 30) χ^2^_1_ = 0.47, *P* = 0.49	13.1 ± 0.06 (n = 24) χ^2^_1_ = 0.06, *P* = 0.8	6.3 ± 0.4 (n = 24) χ^2^_1_ = 0.16, *P* = 0.69
**25°C Control**	89.7 (N = 39)	13.4 ± 0.09 (n = 35)	8.1 ± 0.2 (n = 35)
**36°C Heat-stressed**	87.2 (N = 39) χ^2^_1_ = 0.0145, *P* = 0.90	13.5 ± 0.1 (n = 34) χ^2^_1_ = 0.14, *P* = 0.71	8.1 ± 0.2 (n = 34) χ^2^_1_ = 0.04, *P* = 0.85
**25°C Control**	86.4 (N = 52)	13.3 ± 0.07 (n = 45)	8.4 ± 0.2 (n = 45)
**38°C Heat-stressed**	90.4 (N = 52) χ^2^_1_ = 0.0435, *P* = 0.83	14.7 ± 0.08 (n = 47) **χ^2^_1_ = 93.66, *P* < 0.001**	7.0 ± 0.3 (n = 47) **χ^2^_1_ = 16.4, *P* < 0.001**

Data are presented with mean ± SEM.

**Fig 2:**
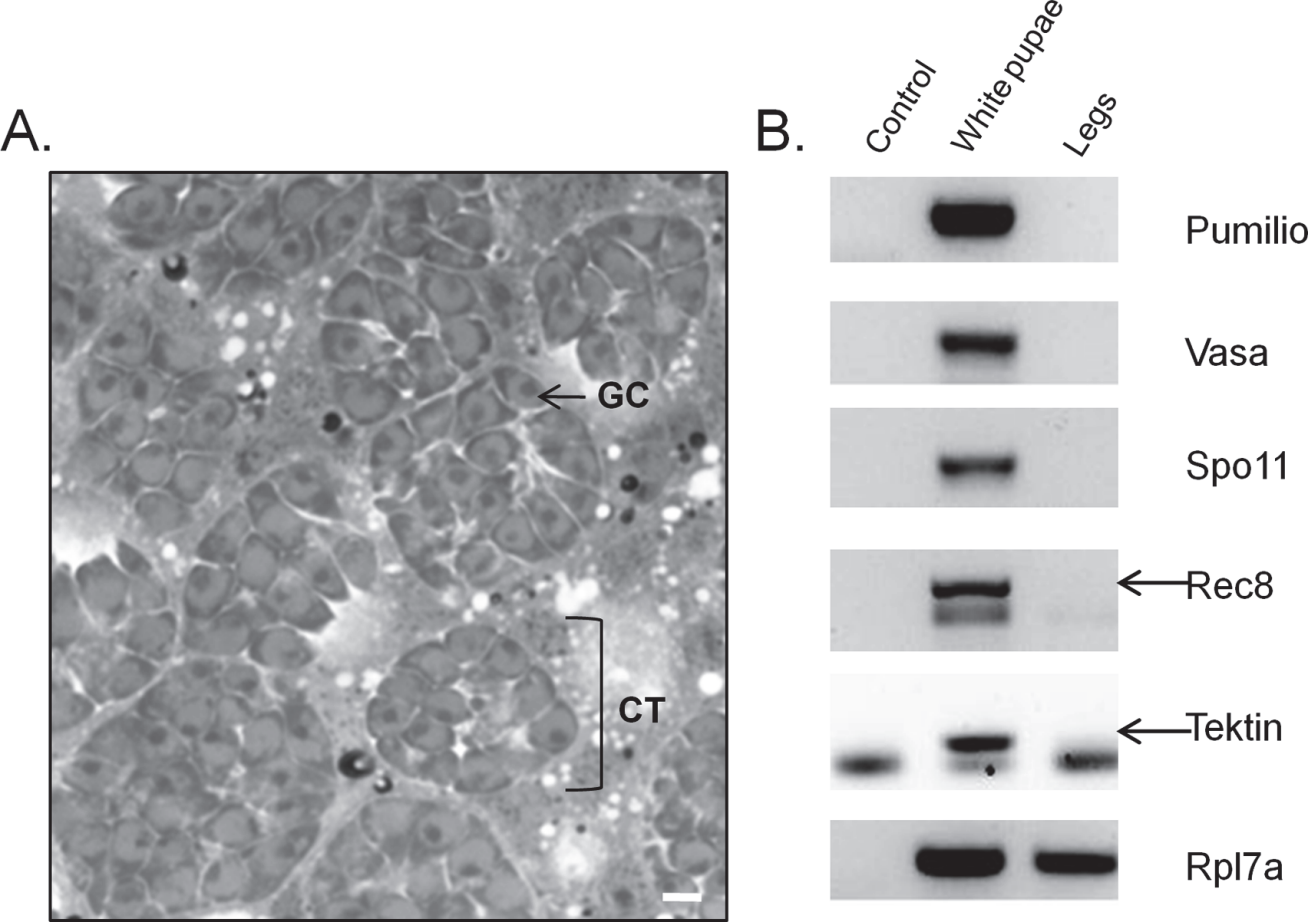
Histological examination of testes at the early pupal stage. A) Morphology of germ cells in cysts from testes. GC = Germ Cell, CT = Cyst. Bar = 10μm. B) mRNA expression of non-differentiated and meiotic germ cells from the early pupal stage and legs.

We investigated whether spermatozoa were present in male testes as early as the first pupal stage (i.e. at the time the heat stress was applied). Inside the testes, germ cells were observed to be arranged in cysts encapsulated in a thin wall (Fig. 2A). Histological examinations of white pupae testes revealed that all cysts contained the same spherical germ cell types with no elongated forms such as spermatids or spermatozoa. The cytoplasm of these germ cells was spherical and contained a large nucleus, a characteristic structure of primary spermatocyte germ cells. Genes specific to undifferentiated germ cells (Pumilio, Vasa) and expressed during meiosis (Spo11, Rec8) or the spermiogenesis process (Tektin) were present in white pupae of control males (Fig. 2B).

**Fig 3:**
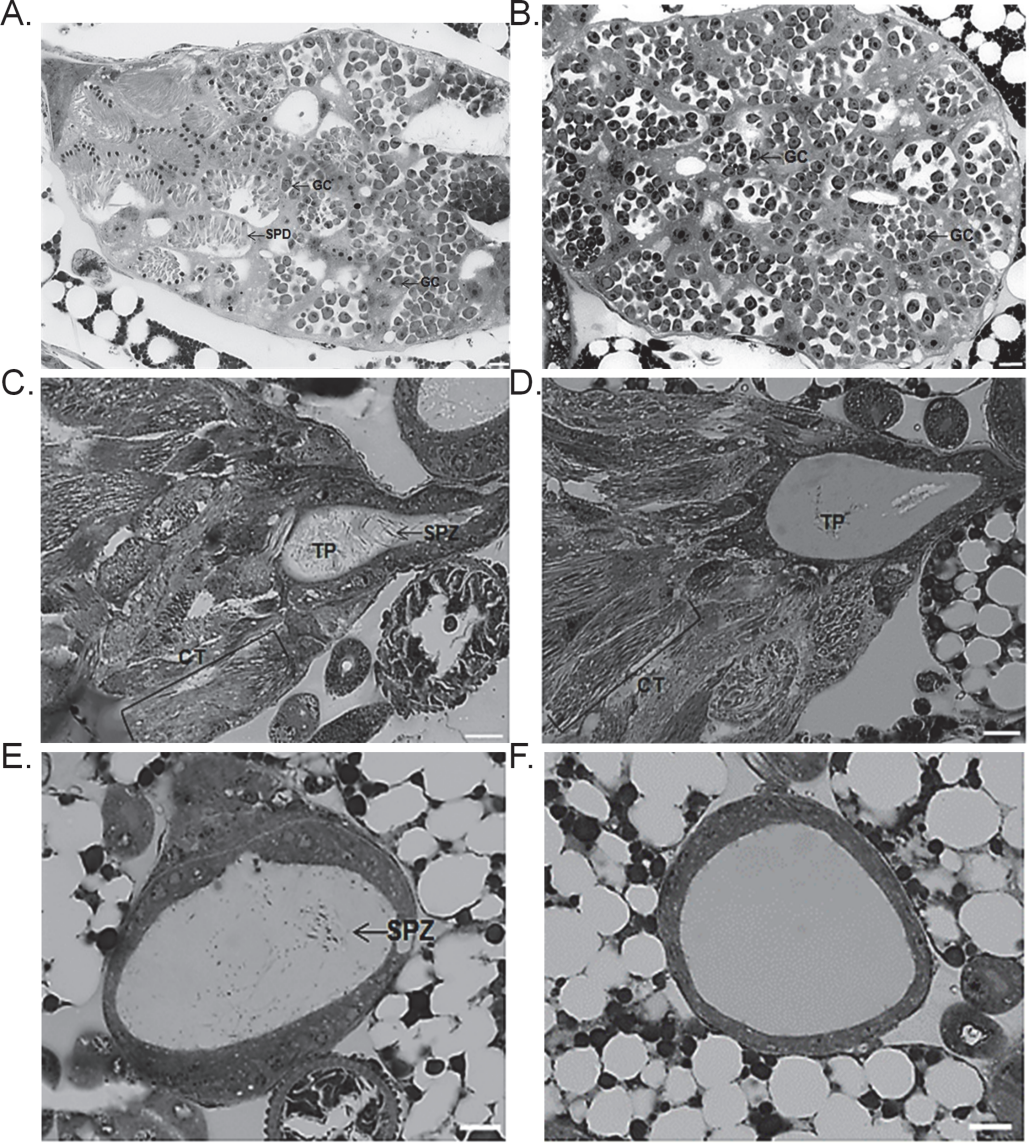
Morphology of the reproductive tract of control and heat-stressed males. A) Morphology of germ cells in cysts from control testes at the second pupal stage. B) Morphology of germ cells in cysts from heat-stressed testes at the second pupal stage (after heat stress). C) Morphology of germ cells in cysts from control testes at the final pupal stage. D) Morphology of germ cells in cysts from heat-stressed testes at the final pupal stage (72 hours after heat stress). D) Morphology of seminal vesicles from control testes at the final pupal stage. E) Morphology of seminal vesicles from heat-stressed testes at the final pupal stage (72 hours after heat stress). GC = Germ Cell, SPD = Spermatid, CT = Cyst, TP = Testis Pocket, SPZ = Spermatozoa. Bar = 10 μm

Histological examination of control testes revealed the presence of cysts containing elongated germ cells such as spermatids and cysts containing multiple spherical non adjacent germ cells. These cells identified as secondary spermatocytes had large nuclei with chromosomes sometimes visible (Fig. 3A). However, heat-stressed male testes harvested after treatment (i.e. second pupal stage) contained only cysts with spherical germ cells with either large nuclei or distinctly visible chromosomes, identified as secondary spermatocytes (Fig. 3B). Testes of control and heat-stressed males at the final pupal stage (i.e. 72 hours after heat stress) revealed that all cysts contained only elongated germ cells (Fig. 3C-D). However, the testis pocket at the base of the control testes contained individual spermatozoon which was not the case for the heat-stressed testes. Moreover, spermatozoa were present in control but not in heat-stressed seminal vesicles (Fig. 3E-F). These observations were in line with the number of spermatozoa counted in seminal vesicles at the final pupal stage of control males, (1799 ± 220.4 spermatozoa; mean ± SEM; n = 8) and of heat-stressed males (no spermatozoa; n = 8).

We quantified the male sex pheromone (4R,5S)- and (4R,5R)-5-hydroxy-4-decanolides (HDL) which is produced by the different categories of males. We found no difference (U = 87.5, *P* = 0.89, n = 13 for both categories) in the quantity released by 36°C heat-stressed (0.33 ± 0.11 ng/male, mean ± SEM) and control males (0.34 ± 0.13 ng/male, mean ± SEM). Similarly, the 36°C heat stress applied during male development had no significant effect on the duration of the different courtship behaviors compared to control males: courtship duration (25°C: 19.2 ± 2.3s, 36°C: 22 ± 2.0s, U = 314, *P* = 0.20), copulation (25°C: 17.4 ± 0.8s, 36°C: 18.7 ± 0.9s, U = 310, *P* = 0.18) and post-copulatory durations (25°C: 17.1 ± 1.0s, 36°C: 17.0 ± 1.1s, U = 393, *P* = 1).

### Consequences of male subfertility on female reproductive strategies

**Fig 4:**
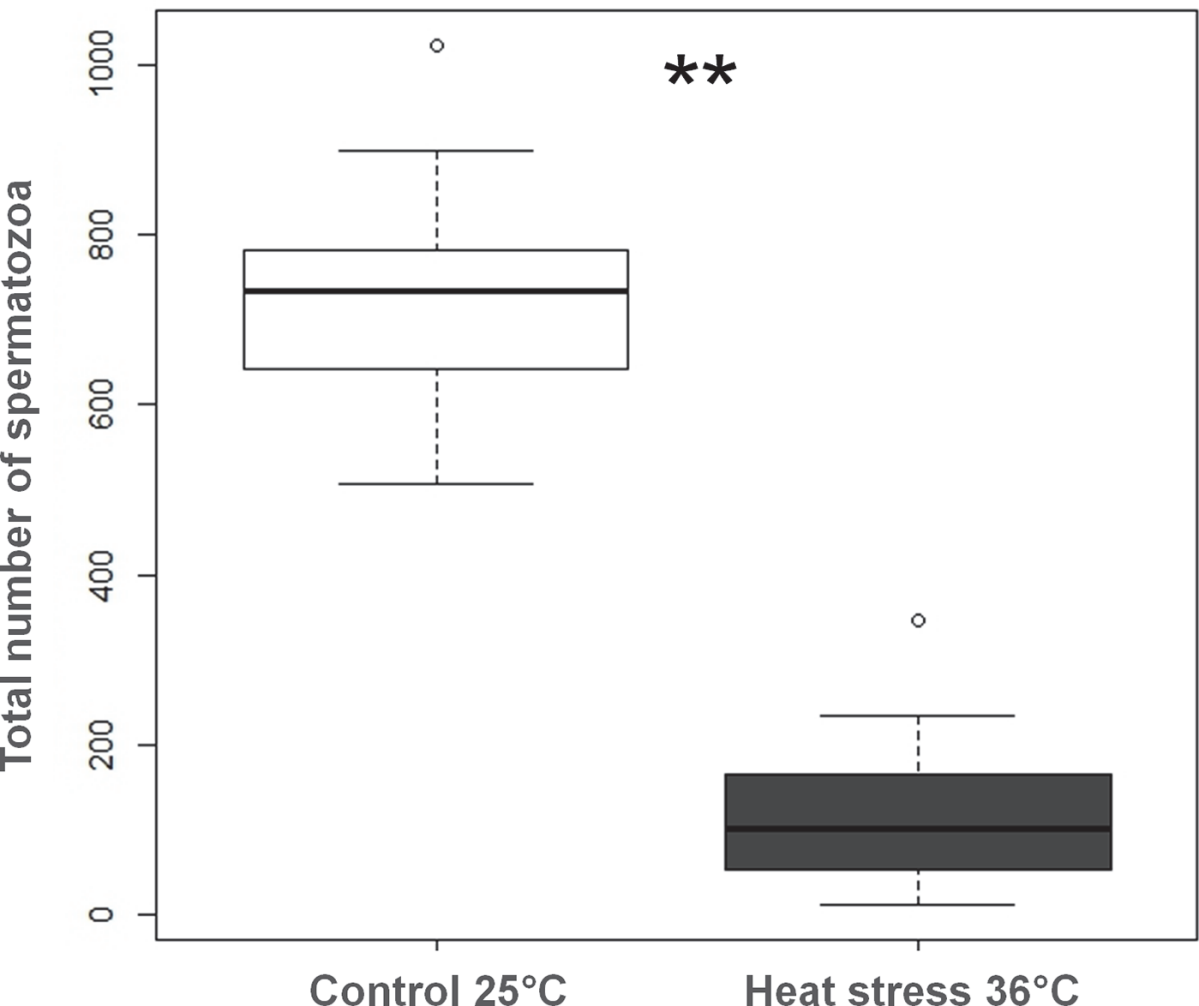
Total number of spermatozoa in female spermatheca after a single mating with either a control or a 36°C heat-stressed male (median with lower and upper quartiles). ** indicates a significant variation between heat-stressed and control males at α = 0.01 (Mann-Whitney, U = 0, N1 = 13, N2 = 13, *P* < 0.01).

Heat stress had a strong impact on the number of spermatozoa transferred to females. After a single copulation with a control male, on average 750 spermatozoa were counted in female spermatheca compared to144 after copulation with a 36°C heat-stressed male. This represents a decrease of 79% (Fig. 4).

**Fig 5:**
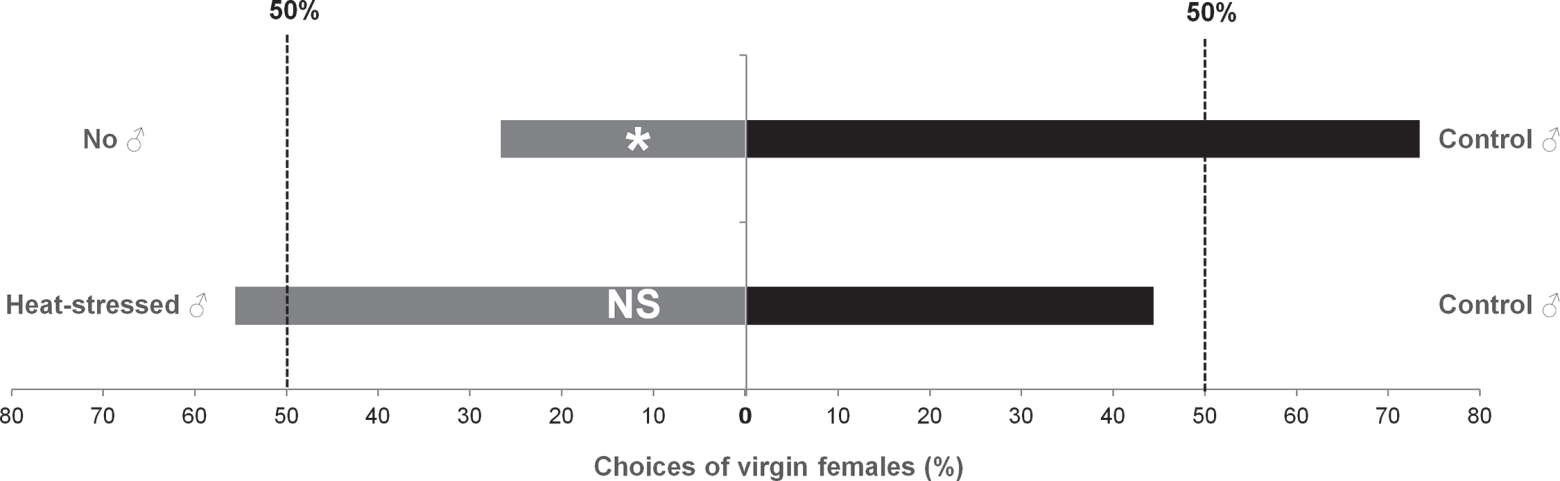
Behavioral response of virgin females to odors of control male versus no male (χ²_1_ = 3.2667, *P* = 0.041, N = 15) or control male versus 36°C heat-stressed male (χ²_1_ = 0.2222, *P* = 0.64, N = 18) in a still-air Y-tube olfactometer. * indicates a significant difference at α = 0.05.

To investigate whether females could recognize and distinguish heat-stressed and control males on the basis of their odors, a female mate choice test was conducted. In a Y-tube olfactometer, virgin females were able to discriminate the presence of control males from no male (Fig. 5). However, in a choice test between 36°C heat-stressed and control males, virgin females did not show any preference between males.

We then investigated whether females that had received a lower sperm quantity from heat-stressed males remated more frequently in order to increase their sperm stock. Our data showed that females which mated with a 36°C-heat stressed male were not more likely to remate than those mated with a control male. Indeed, after oviposition, 27.9% of females mated with a heat-stressed male (n = 42) and 44.4% of those mated with a control male (n = 45) accepted a second partner (χ²_1_ = 0.81, *P* = 0.37).

**Fig 6:**
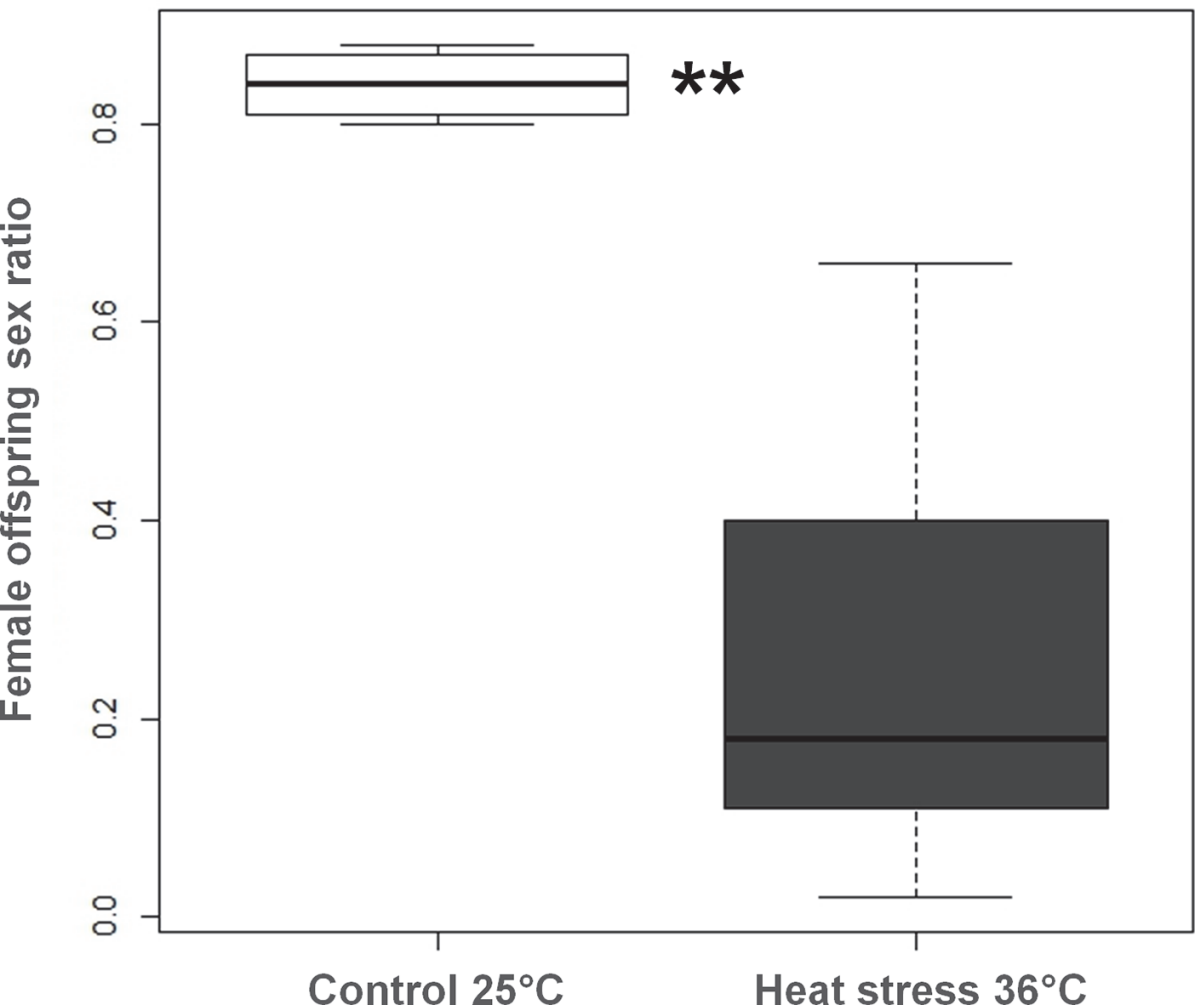
Lifetime offspring sex ratio (proportion of females) produced by mated females with either a control or a heat-stressed male (median with lower and upper quartiles). ** indicates significant variation between female offspring of females mated with heat-stressed or control males at α = 0.01 (Mann-Whitney, U = 272, N1 = 20, N2 = 17, *P* < 0.01).

Consequently, the lifetime offspring sex ratio (proportion of males and females) was reversed in 36°C-treated males compared to controls (Fig. 6). The offspring sex ratio of females mated with a control male was strongly female-biased (84%), whereas that of females mated with a 36°C-treated male was strongly male-biased (26% of females).

## Discussion

The present study revealed that heat stress applied during male juvenile development caused a significant temperature-dependent decrease in sperm stock. This is in line with previous studies on parasitoids which have reported a reduction in sperm stock in adult males exposed to different stresses (e.g. temperature, chemical or eating disorders) during development [5,45–47]. A temperature of 36°C applied for 24h during the early pupal stage had no consequence on the emergence rate, longevity or weight (data not shown) of males but seemed to disrupt their spermatogenesis resulting in a reduced number of spermatozoa available in adult males.

Our results therefore show that heat stress acted on sperm production and not directly on mature sperm. Indeed histological analyses show that during the early pupal stage, only primary spermatocytes were present [48,49]. This observation supports the results obtained by RT-PCR as the mRNA expression of genes involved in non-differentiated and meiosis processes were detected [50]. In mammals, spermatocytes and round spermatids are germ cells predominantly sensitive to temperature [51,52]. Indeed, elevated temperatures induce activation of the heat shock transcription factor 1 (HSF1), which in somatic cells, leads to heat-shock protein synthesis and cryoprotection; and in spermatocytes induces caspase-3 dependent apoptosis [53]. Thus, it could be hypothesized that spermatocytes undergo apoptosis during nymphal development possibly responsible for spermatozoa loss observed in emerged males. In any case, a 24-hour delay in testes pocket and seminal vesicle filling was observed following heat-stressing. It is possible that, during the 24 hours of exposure to heat, spermatogenesis was temporarily interrupted: spermatocyte differentiation would have been paused and then restarted. During stress, energy resources used for the costly production of sperm can indeed have been cut off, being used instead for survival. As most parasitoid species are incapable of *de novo* lipogenesis [54], only a limited quantity of lipid is available and can either be allocated to gamete production or survival.

As only one wave of spermatogenesis occurs, the consequence of sperm depletion during the pupal stage is therefore irreversible and permanent: heat-stressed males have fewer spermatozoa in their seminal vesicles during adulthood (data not shown). Consequently, male capacities to inseminate females and distribute their sperm via multiple matings would be limited and we can predict a decrease in male fitness. Moreover, the other mating traits we measured seemed unaffected by heat stress, making heat-stressed males potentially unidentifiable by females. Indeed, courtship duration and behaviors of heat-stressed males were similar to those of control males, as has been observed after a cold shock [55] demonstrating that neither hot nor cold temperatures affect male courtship behaviors. Additionally, the sex pheromone titers (HDL) produced by males were not affected by heat stress and females could therefore not differentiate males on that basis. In contrast, Ruther and coworkers found that *N*. *vitripennis* females can discriminate several levels of sperm-depleted males after multiple matings based on the reduction of HDL titers [39]. Our results therefore suggest that pheromone release was not directly correlated with sperm production but with mating numbers. As a result, the probability of heat-stressed males attracting females would be the same as for control males. Moreover, our results show that females which mated with heat-stressed sperm-limited males did not remate more frequently and consequently did not compensate the lack of sperm transferred.

The consequence of heat-stress on male sperm production and thus on sperm available for females can have marked effects on the composition of the next generation, and potentially on the population dynamics of these wasps. Indeed, in haplodiploid species the ability of females to produce an optimal offspring sex ratio in a lifetime depends on the quality and quantity of sperm received by females during copulation [56]. In our study the quality of sperm transferred to females seemed unaltered by heat stress as some daughters were still produced (133 sperm stored in spermatheca of female mated with a stressed male; 107 daughters produced by each of these female in their lifespan). This high sperm efficiency has been previously reported in another ectoparasitoid species: *Dinarmus basalis* (Hymenoptera: Pteromalidae) [57]. Nevertheless, the reduced sperm quantity seemed to strongly affect their primary sex-ratio. Females mated with a heat-stressed male laid a similar number of eggs as females mated with a control male, but as they did not possess a sufficient number of spermatozoa to fertilize all of them, in their lifetime they would produce a suboptimal offspring sex ratio with a greater proportion of males (produced through arrhenotokous parthenogenesis). This modification in sex ratio resulting in a male rather than a female bias decreases the reproductive success of females mated with heat-stressed males because only daughters are able to fly and disperse to reproduce on new host patches [23,58]. Moreover based on Hamilton’s theory, we can predict that competition between their flightless sons to inseminate females would increase, as a minimum number of males are necessary to inseminate all daughters [23,58]. Another potential consequence is that the next generation could have a lower parasitic activity because only females lay their eggs in hosts and these changes in sex ratios may affect the population dynamics [59]. Modification of the population structure could be even more affected if heat stress also caused damage to the normal functioning of the reproductive system in females. Indeed, it has previously been shown that high temperatures could disrupt oocyte and ovarian development in females and lead to a decrease in egg production [31]. Consequently, in the future it could be interesting to study the effects of heat stress on female reproduction in *Nasonia vitripennis*.

## Conclusion

This work thus demonstrates, as previous studies [60,61], that it is important to control the temperature in mass production of parasitoids to allow more efficient use of wasps in biological control. Furthermore, such a heat-stress effect could also be dramatic in other hymenoptera species of agronomic interest, such as *Apis mellifera* which also shows synchronized spermatogenesis [62]. The potential reduction in the number of daughters produced by *A*. *mellifera* queens *could* affect colony persistence as worker bees are only females. Subsequently, the associated loss of pollination would have important negative ecological and economic impacts on for instance maintenance of wild plant diversity and crop yield, respectively. For species with one wave of spermatogenesis but no haplo-diploidy, the consequences would still be visible as it would reduce the total number of offspring produced. Therefore, *N*. *vitripennis* is an interesting model to study the mechanisms and consequences of subfertility induced in species with an agronomic importance such as bees. As the *Nasonia* genome and genetic tools are available in this species, comparative studies may provide new ways to understand mechanisms of male subfertility in vertebrates and invertebrates.
